# Timing matters: sex differences in treatment limitation decisions in intensive care

**DOI:** 10.1186/s13054-026-06139-x

**Published:** 2026-06-19

**Authors:** Simon A. Amacher, Pimrapat Gebert, Valentina Tröster, Nidaa Mikail, Adriana Vinzens, Vera Regitz-Zagrosek, Ketina Arslani, Julie Helms, Hamid Merdji, Micah T. Long, Sabina Hunziker, Martin Lohri, Pascale Grzonka, Sebastian Berger, Alexa Hollinger, Karin Wildi, Bernd Yuen, Mark Kaufmann, Raoul Sutter, Martin Siegemund, Catherine Gebhard, Caroline E. Gebhard

**Affiliations:** 1 Intensive Care Unit, Department of Acute Medicine, University Hospital Basel, Basel, Switzerland; 2 Department of Anesthesiology and Critical Care, Faculty of Medicine, Medical Center University of Freiburg, University of Freiburg, Freiburg, Germany; 3 Institute of Biometry and Clinical Epidemiology, Charité-Universitätsmedizin Berlin, Berlin, Germany; 4 Department of Nuclear Medicine, University Hospital Zurich, Zurich, Switzerland; 5 Center for Molecular Cardiology, University of Zurich, Schlieren, Switzerland; 6 Institute of Gender in Medicine (GiM), Charité - Universitätsmedizin Berlin, Berlin, Germany; 7 Department of Cardiology, University Hospital Basel, Basel, Switzerland; 8 Faculté de Médecine, Service de Médecine Intensive-Réanimation, Université de Strasbourg (UNISTRA), Hôpitaux universitaires de Strasbourg, Nouvel Hôpital Civil, Strasbourg, France; 9 INSERM (French National Institute of Health and Medical Research), UMR 1260, Regenerative Nanomedicine (RNM), FMTS, Strasbourg, France; 10 Department of Anesthesiology and Critical Care, University of Wisconsin Hospitals and Clinics, Madison, USA; 11 Medical Communication and Psychosomatic Medicine, University Hospital Basel, Basel, Switzerland; 12 University of Basel, Basel, Switzerland; 13 Anesthesiology, University Hospital Basel, Basel, Switzerland; 14 Interdepartmental Division of Critical Care, University Health Network, Toronto, Canada; 15 Intensive Care Unit, Cantonal Hospital Aarau, Aarau, Switzerland; 16 Intensive Care Unit, Stadtspital Zürich Triemli, Zurich, Switzerland; 17 Department of Neurology, University Hospital Basel, Basel, Switzerland; 18 Department of Cardiology, Bern University Hospital, Inselspital, University of Bern, Bern, Switzerland

**Keywords:** Treatment limitation, Intensive care unit, Sex, Gender, End-of-life decision-making, Withholding and withdrawal of life-sustaining therapies.

## Abstract

**Background:**

Sex differences in intensive care treatment and mortality are well documented, but the timing of decisions to limit treatment remains unclear. We investigated whether sex differences in decisions to limit treatment arise at ICU admission or during the ICU stay.

**Methods:**

Nationwide cohort study using the Swiss Minimal Dataset for Intensive Care Units, including adult (≥ 18 years) ICU admissions between 2016 and 2024. Two adjusted logistic regression models assessed treatment limitations documented at ICU admission and those occurring later among patients admitted without limitations.

**Results:**

Among 654,660 ICU stays, treatment limitations at admission were more common in women than men (12.0% vs. 8.6%), whereas rates during the ICU stay were similar (5.5% vs. 5.5%). Female sex was independently associated with limitations at admission (aOR 1.26, 95% CI 1.24–1.28) but only weakly associated with later limitations (aOR 1.10, 95% CI 1.08–1.13). Differences at admission varied by diagnosis and were most pronounced in trauma and cardiovascular conditions. Women more often had ceiling-of-care decisions and documented patient wishes, whereas men more frequently underwent withdrawal of life-sustaining therapies and physician-driven decisions. Mortality was highest with limitations at ICU admission and lowest without limitations, with minimal sex differences within categories.

**Conclusions:**

In Switzerland, sex differences in treatment limitations occur mainly at ICU admission and vary across diagnoses. These findings suggest that differences may reflect early triage heuristics, societal norms influencing advance care planning, and potential implicit biases under prognostic uncertainty. Structured goal-of-care discussions at ICU admission may help promote consistent and equitable decision-making.

**Graphical abstract:**

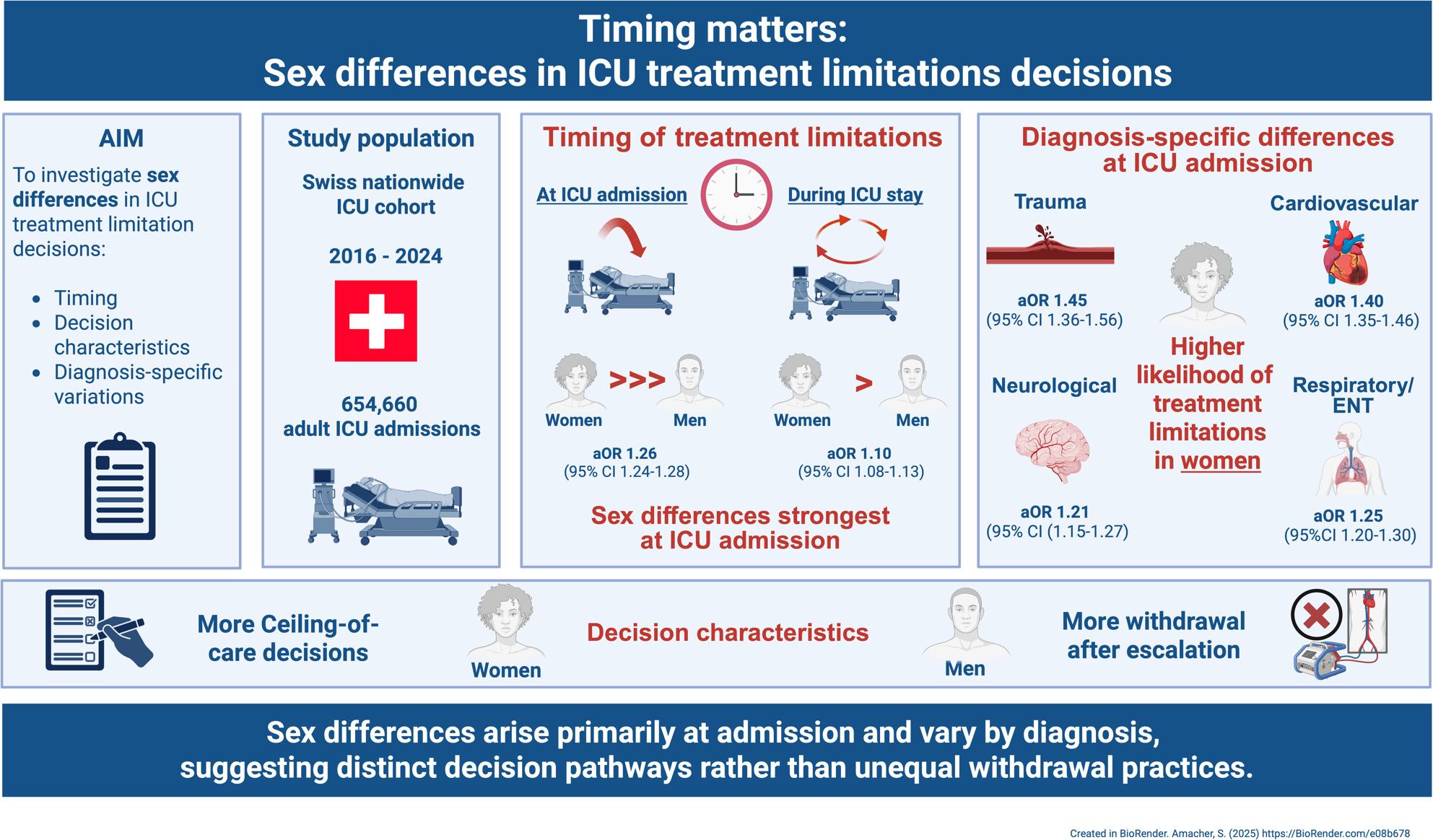

**Supplementary Information:**

The online version contains supplementary material available at 10.1186/s13054-026-06139-x.

## Background

Decisions to withhold or withdraw life-sustaining therapies are a central component of modern intensive care medicine [1]. These decisions influence admission thresholds, escalation strategies, and end-of-life care, thereby shaping the clinical trajectory of critical illness. They require integration of prognosis, patient preferences, and medical judgement, often under substantial uncertainty and time pressure.

Treatment limitation decisions are closely linked to mortality in intensive care, as many deaths in the ICU are preceded by withholding or withdrawal of life-sustaining therapies [1–3]. Consequently, differences in ICU mortality between patient groups may partly reflect differences in decision-making processes rather than disease severity alone. This is particularly relevant in the context of reported sex differences in ICU outcomes [4]. 

A growing body of literature demonstrates sex differences in intensive care treatment and outcomes [5]. Women receive less invasive organ support than men after adjustment for illness severity, and outcome differences vary by age and diagnostic category [5–7]. Prior registry analyses have further documented systematic differences in ICU care processes and outcomes between men and women [8]. These observations suggest that disparities are context-dependent and may be most pronounced when prognosis is uncertain. However, previous work has largely focused on treatment intensity or mortality rather than on the timing of treatment limitations.

Treatment limitations may occur before or at ICU admission or later during the ICU stay, representing distinct decision contexts. Early decisions are frequently made under prognostic uncertainty, whereas later decisions increasingly incorporate clinical evolution and treatment response. Understanding whether sex differences in ICU care arise at the time of ICU entry or during ongoing treatment is essential for interpreting differences in treatment intensity and mortality and for identifying where in the care pathway potential inequities emerge.

We therefore investigated whether sex differences in the timing of treatment limitation decisions occur at ICU admission or evolve during the ICU stay, after adjustment for age, illness severity, diagnosis, and institutional context, thereby distinguishing admission-level decision processes from more prognosis-driven decisions during intensive care.

## Methods

### Study design and data source

This study used data from the Swiss Minimal Dataset for Intensive Care Units (MDSi), a nationwide prospective registry maintained by the Swiss Society of Intensive Care Medicine. Reporting to this registry is mandatory for all accredited ICUs in Switzerland [9]. During the study period, 75 accredited adult ICUs contributed data continuously. For each ICU admission, a standardized minimal dataset is recorded, including demographics, admission pathway, diagnostic category, illness severity scores, institutional characteristics, and discharge information, as reported previously [8, 10–12]. After automated plausibility checks and local verification, data are anonymized and transferred to a central database for quality control and research use [9]. The study followed STROBE and SAGER reporting guidelines.

## Study population

All adult patients (≥ 18 years) admitted to certified Swiss ICUs between January 1, 2016, and December 31, 2024, were included. The start of the study period was chosen because structured documentation of treatment limitation decisions became available in the registry 2016 onwards. Patients were included if treatment limitation status was documented, allowing classification of the timing, degree, and reason for limitation and differentiation between decisions present at ICU admission and those occurring later during the ICU stay. To avoid duplicate counting of the same clinical episode, ICU readmissions within 48 h (*n* = 15’300) were excluded from the analysis. Patients admitted with sex-specific diagnoses (e.g., obstetric and gynecological conditions) were excluded to allow comparison of treatment limitation decisions between women and men across comparable clinical conditions. Sex assigned at birth, as recorded in the registry, was used as the exposure variable and categorized as women and men. The final study cohort was derived after application of predefined inclusion and exclusion criteria (Fig. 3).

## Treatment limitation definitions

Treatment limitation decisions were classified according to registry definitions [2]. The timing of limitation was categorized as present during the process of ICU admission, occurring during the ICU stay, or documented at ICU discharge. Limitations at ICU admission were defined as those documented during the admission process and may reflect pre-existing patient directives or decisions made prior to or at the time of ICU admission (e.g., in the emergency department), whereas limitations occurring later were classified as during the ICU stay. The degree of limitation reflected either time-limited treatment (unfavorable or unclear prognosis in long-term outcome, but favorable in short term, e.g. treatment of temporary organ dysfunction), limitation in treatment content (poor medium or long-term prognosis based on comorbidities, age, functional status), or withdrawal of life-sustaining therapies with transition to palliation. The registry also captured whether the decision was based on the patient’s wishes (presence of health directives delivered in written or oral form), a surrogate decision maker (in case of absent or unknown patient’s directives), or a medical/physician-driven futility decision (clinical deterioration despite full intensive care, with no reasonable prospect that continued treatment would provide meaningful benefit or allow return to an appropriate living environment).

## Covariates

Covariates were selected a priori based on clinical relevance and prior literature on disparities in ICU outcomes [8, 10–12]. These included age, admission diagnosis category, origin prior to ICU admission, illness severity using the Simplified Acute Physiology Score II (SAPS II) and the Nine Equivalents of Nursing Manpower Use Score (NEMS) measured within the first eight hours. ICU type (medical, surgical, interdisciplinary) and hospital level of care (academic, large/cantonal, regional). These variables represent baseline prognosis and triage context at ICU admission rather than downstream treatment intensity.

## Outcomes

Two complementary outcomes were defined to distinguish early admission decisions from later prognosis-dependent decisions: (1) Treatment limitation present at ICU admission and (2) newly documented treatment limitation during the ICU stay or at ICU discharge among patients without a limitation at admission.

### Statistical analysis

Continuous variables were reported as mean with standard deviation or median with interquartile range as appropriate, and categorical variables as counts and percentages. Missing data were assessed for all variables. No missing values were identified in the analytic dataset; therefore, no imputation or additional missing data handling was required. Baseline differences were described using standardized mean differences (SMD). Two multivariable logistic regression models assessed the association between sex and treatment limitations: one for limitations at ICU admission and one for limitations during the ICU stay among patients admitted without initial limitations. This staged approach was chosen to separate admission triage decisions from decisions informed by thorough clinical evaluation during the ICU stay. Models were adjusted for predefined covariates, including age, illness severity, diagnosis category, admission characteristics, ICU type, and hospital category to account for institutional differences. Variables reflecting downstream treatment intensity were intentionally not included to prevent over-adjustment. In a sensitivity analysis, patient origin prior to hospital admission (e.g., home versus nursing facility) was included as a proxy for baseline functional status. To explore whether the association between sex and treatment limitation varied across clinical contexts, interaction between sex and diagnosis category was performed. Model performance and parsimony were compared using the Bayesian information criterion, with the non-interaction model retained as the primary analysis unless a clear improvement in model fit was observed. Adjusted odds ratios (ORs) with 95% confidence intervals (CIs) were reported. Two-sided p-values < 0.05 were considered statistically significant. Analyses were performed using Stata MP/18 (StataCorp, 2023, College Station, Texas, USA).

## Results

### Study population

During the study period from 2016 to 2024, 681,922 ICU admissions met eligibility criteria. After applying predefined inclusion and exclusion criteria, 654,660 ICU stays (259,220 women [39.6%] and 395,440 men [60.4%]) were included in the primary analysis of treatment limitations documented at ICU admission. For analyses of treatment limitations occurring during the ICU stay or at discharge, patients with treatment limitations already present at ICU admission were excluded, resulting in a cohort of 589,500 ICU stays (228,091 women [38.7%] and 361,409 men [61.3%]). Patient selection is shown in Fig. 3.

### Frequency of treatment limitation in the overall population

Treatment limitations were documented in a substantial proportion of patients and occurred more often in women than in men (18.1% vs. 14.6%, *p* < 0.001, SMD 0.094 Fig. 4A). When stratified by diagnostic category, a higher frequency of treatment limitations in women was observed across most admission diagnoses, with the largest differences observed in trauma and cardiovascular conditions and minimal or no differences in sepsis/septic shock and metabolic/endocrine conditions (Fig. 4B).

### Baseline characteristics of patients with treatment limitations at ICU admission

Patients with treatment limitations at ICU admission differed substantially from those without limitations (Table 1). They were markedly older (mean age 78.0 vs. 63.7 years) and had higher illness severity scores at admission (SAPS II 41.9 vs. 30.4). Treatment limitations at admission were more frequent among emergency admissions and medical ICUs, and less frequent among elective surgical admissions. Across diagnostic categories, treatment limitations at ICU admission were most frequent in sepsis, respiratory, and neurological diagnoses and less frequent in cardiovascular and metabolic conditions (Table 1). Patients in whom treatment limitations were introduced during the ICU stay or at ICU discharge had intermediate age (mean 72.4 years) but the highest illness severity at admission (SAPS II 55.8). Table 1 describes group composition and should not be interpreted as risk differences; the association between sex and treatment limitation was evaluated in regression analyses.

**Table 1:** Baseline characteristics of the study population according to the timing of treatment limitation

	No treatment limitations at ICU admission (*n* = 549,737)	Treatment limitations at ICU admission (*n* = 65,160)	Treatment limitations during ICU stay or at ICU discharge (*n* = 39,763)
**Demographics**			
Age, years, mean (SD)	63.7 (16.2)	78.0 (11.1)	72.4 (12.9)
Sex, n (%)			
Female	212,205 (38.6%)	31,129 (47.8%)	15,886 (40.0%)
Male	337,532 (61.4%)	34,031 (52.2%)	23,877 (60.0%)
**Illness severity at ICU admission**			
SAPS II score, mean (SD)	30.4 (15.2)	41.9 (16.5)	55.8 (20.4)
NEMS first 8 h, mean (SD)	20.7 (9.2)	20.9 (8.6)	27.5 (10.4)
**Admission type and origin**			
Pre-hospital patient origin, n (%)			
From home	447,575 (81.4%)	49,012 (75.2%)	27,832 (70.0%)
Other hospital	54,127 (9.8%)	7,027 (10.8%)	6,271 (15.8%)
Nursing home	3,199 (0.6%)	2,052 (3.2%)	537 (1.3%)
Other	44,836 (8.2%)	7,069 (10.8%)	5,123 (12.9%)
Admission type, n (%)			
Elective	182,260 (33.2%)	8,283 (12.7%)	4,050 (10.2%)
Emergency	367,477 (66.8%)	56,877 (87.3%)	35,713 (89.8%)
**ICU and hospital characteristics**			
ICU type, n (%)			
Medical ICU	23,171 (4.2%)	5,511 (8.5%)	2,076 (5.2%)
Surgical ICU	38,145 (6.9%)	2,600 (4.0%)	2,265 (5.7%)
Mixed or other ICU	488,421 (88.9%)	57,049 (87.5%)	35,422 (89.1%)
Hospital category, n (%)			
University hospital	108,045 (19.6%)	9,347 (14.3%)	12,274 (30.9%)
Large/cantonal hospital	157,066 (28.6%)	16,074 (24.7%)	12,189 (30.6%)
Regional hospital/ no categorization	284,626 (51.8%)	39,739 (61.0%)	15,300 (38.5%)
**Primary diagnosis category**, n (%)			
Sepsis/septic shock	21,635 (3.9%)	5,363 (8.2%)	4,500 (11.3%)
Respiratory/ENT diagnoses	66,202 (12.0%)	11,923 (18.3%)	8,304 (20.9%)
Neurological diagnoses	81,456 (14.8%)	11,775 (18.1%)	6,835 (17.2%)
Trauma	31,754 (5.8%)	4,897 (7.5%)	2,192 (5.5%)
Gastrointestinal diagnoses	71,706 (13.0%)	8,567 (13.1%)	4,293 (10.8%)
Cardiovascular diagnoses	184,837 (33.6%)	14,881 (22.8%)	11,013 (27.7%)
Urogenital diagnoses	13,865 (2.5%)	1,376 (2.1%)	416 (1.0%)
Metabolic/endocrine diagnoses	35,767 (6.5%)	2,719 (4.2%)	857 (2.2%)
Other ICU diagnoses	42,515 (7.7%)	3,659 (5.6%)	1,353 (3.4%)
**Risk of treatment limitations by sex**			
	**Total number**	**At ICU admission**	**During ICU stay or at ICU discharge**
**Women**	243,334	31,129 (12.0%)	15,886 (6.1%)
**Men**	371,563	34,031 (8.6%)	23,877 (6.1%)

Values are presented as n (%) for categorical variables and mean (SD) or median (IQR) for continuous variables, as appropriate. The table includes patients with no treatment limitations, treatment limitations documented at ICU admission, and treatment limitations documented during the ICU stay or at ICU discharge. ICU, intensive care unit; IMC, intermediate care unit; ENT, ear, nose and throat; SAPS II, Simplified Acute Physiology Score II; NEMS, Nine Equivalents of Nursing Manpower Use Score

### Timing of treatment limitations

**Fig. 1:**
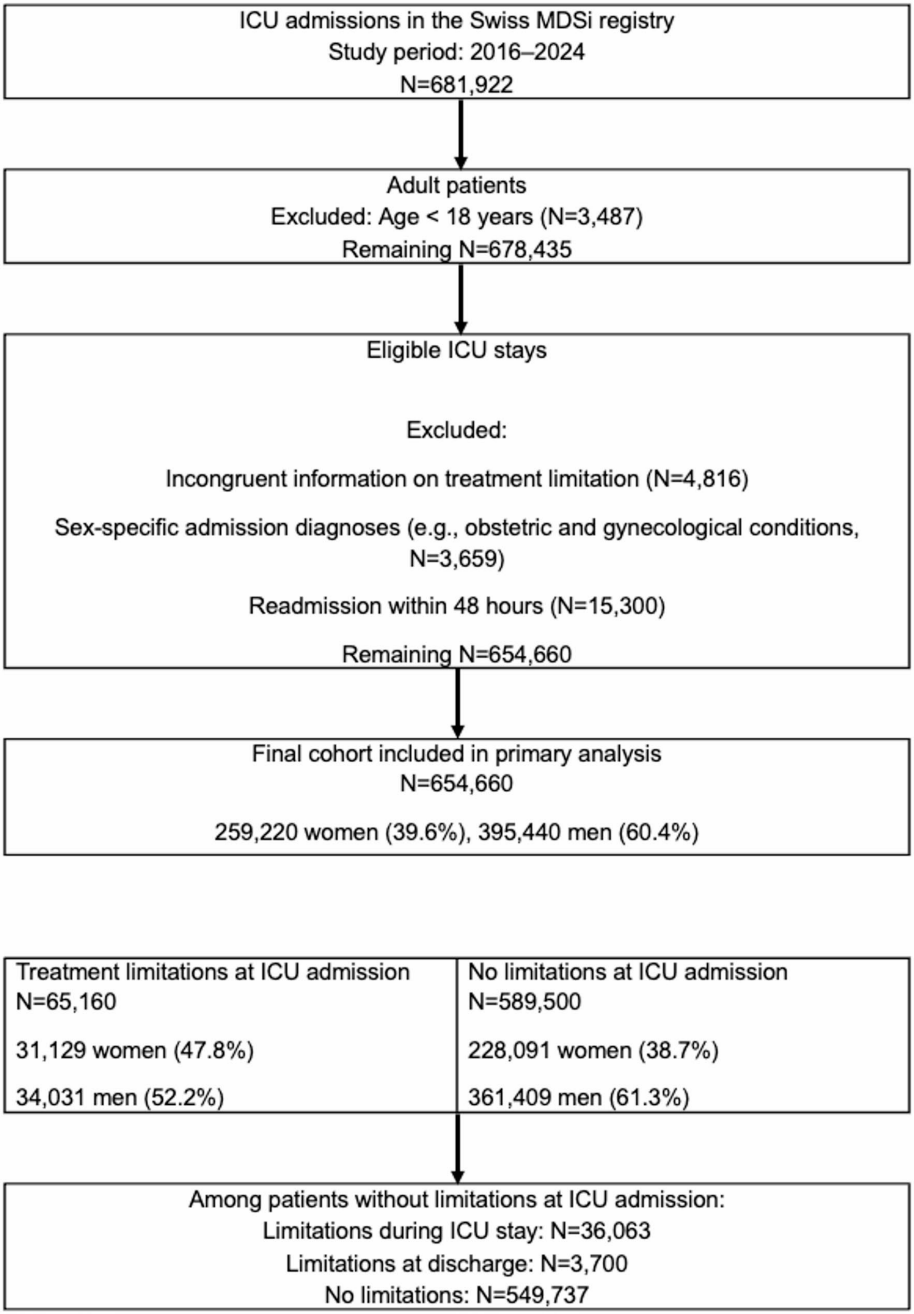
Study flow chart. Flow diagram of patient selection from the Swiss Minimal Dataset for Intensive Care Units (MDSi). Adult ICU admissions were screened according to predefined eligibility criteria and exclusions to derive the final study population analyzed for treatment limitation decisions. ICU, intensive care unit

Sex differences were concentrated at ICU admission (Fig. 1A, Supplementary Table 1A). Treatment limitations at ICU admission occurred more often in women than in men (12.0% vs. 8.6%). In contrast, limitations arising during the ICU stay (5.5% vs. 5.5%) or at ICU discharge (0.6% vs. 0.6%) were similar between sexes (Supplementary Table 1A). Subsequent analyses describe only patients with treatment limitations.

**Fig. 2:**
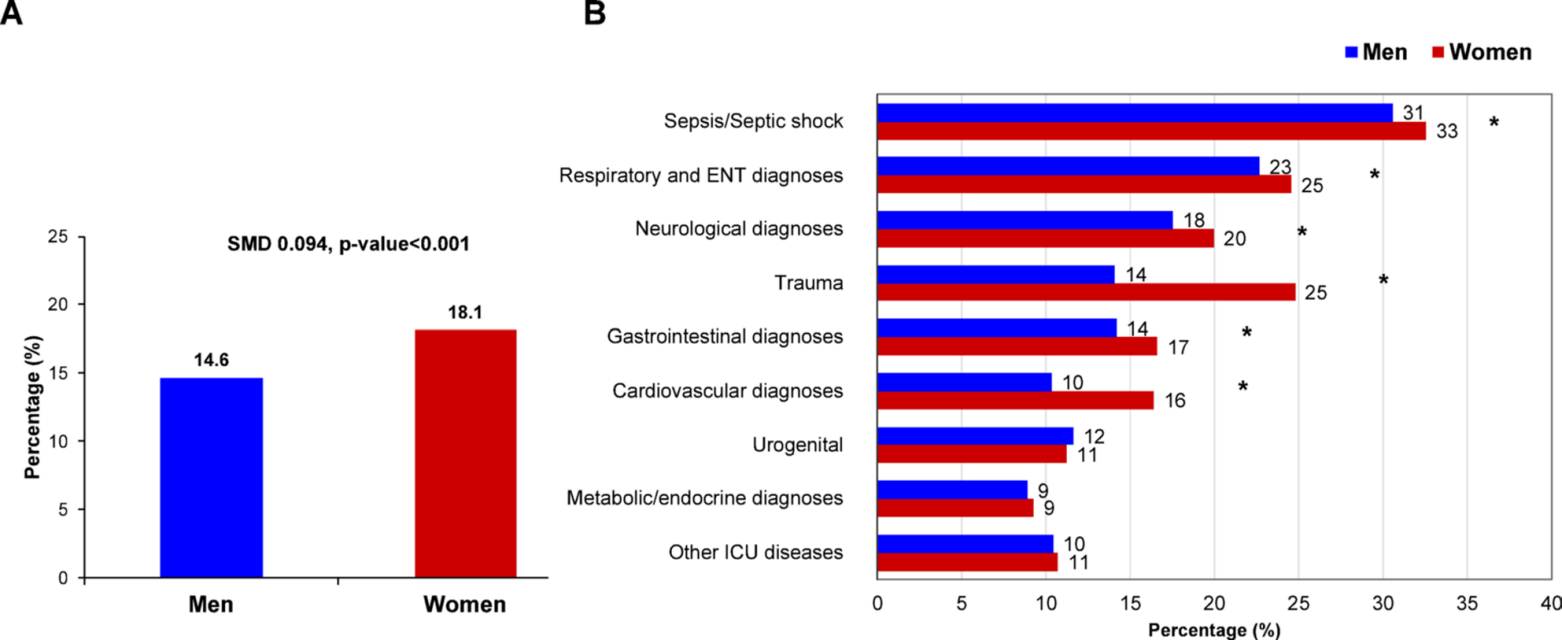
Treatment limitations by sex and diagnostic category. **A**. Percentage of ICU patients with any documented treatment limitation in women and men. **B**. Percentage of ICU patients with treatment limitations stratified by admission diagnostic category. Numbers represent the percentage within each diagnostic group. Asterisks* indicate significant differences based upon a significance level of p-value < 0.05 (two-sided). ENT, ear-nose-throat; ICU, intensive care unit; SMD, standardized mean difference

**Fig. 3:**
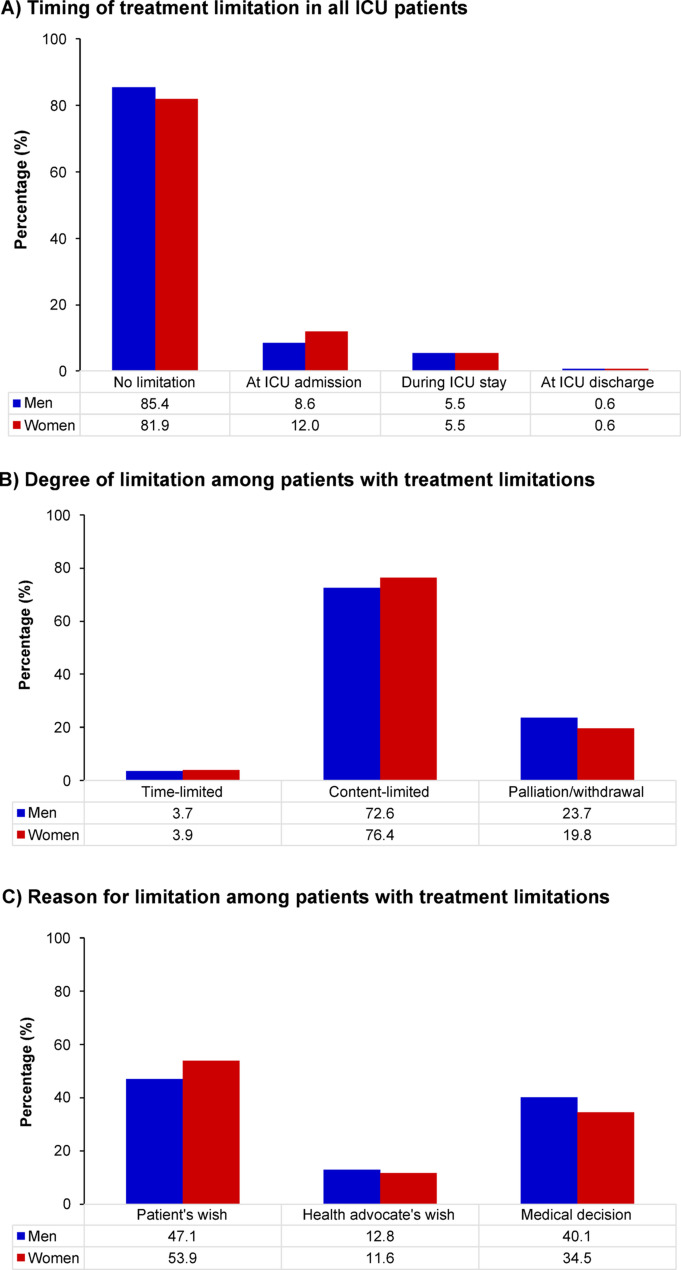
Timing and characteristics of treatment limitation decisions according to sex.** A** Timing of treatment limitations in the overall ICU population (at ICU admission, during ICU stay, or at ICU discharge). **B** Degree of treatment limitations among patients with documented limitations. **C** Documented reason for treatment limitations among patients with documented limitations. Percentages in panels **B** and **C** are calculated within the subgroup of patients with treatment limitations and therefore describe decision characteristics rather than the likelihood of treatment limitations. ICU, intensive care unit

### Characteristics of treatment limitation decisions

Among patients with documented treatment limitations, the characteristics of decisions differed modestly by sex (Fig. 1B **− **C, Supplementary Table 1B). Women more often had limitations defined as ceilings of care, whereas men more often underwent withdrawal of life-sustaining therapies. Patient wishes were documented more frequently in women, while medical reasons/physician-driven decisions were more common in men; surrogate decision-maker involvement was similar between sexes. These analyses are restricted to patients with treatment limitations and therefore describe decision characteristics rather than the likelihood of limitations.

### Multivariable analysis

#### Treatment limitations at ICU admission

After adjustment for age, illness severity, diagnosis, admission context, and institutional characteristics, female sex was associated with a higher likelihood of treatment limitations at ICU admission (aOR 1.26, 95% CI 1.24–1.28, Fig. 2A). Age and illness severity were strong predictors (aOR per 10 years 2.24; per 10 SAPS II points 1.31). Results were unchanged in sensitivity analyses including patient origin prior to hospital admission, with slightly improved model performance (Supplementary Table 2).

**Fig. 4:**
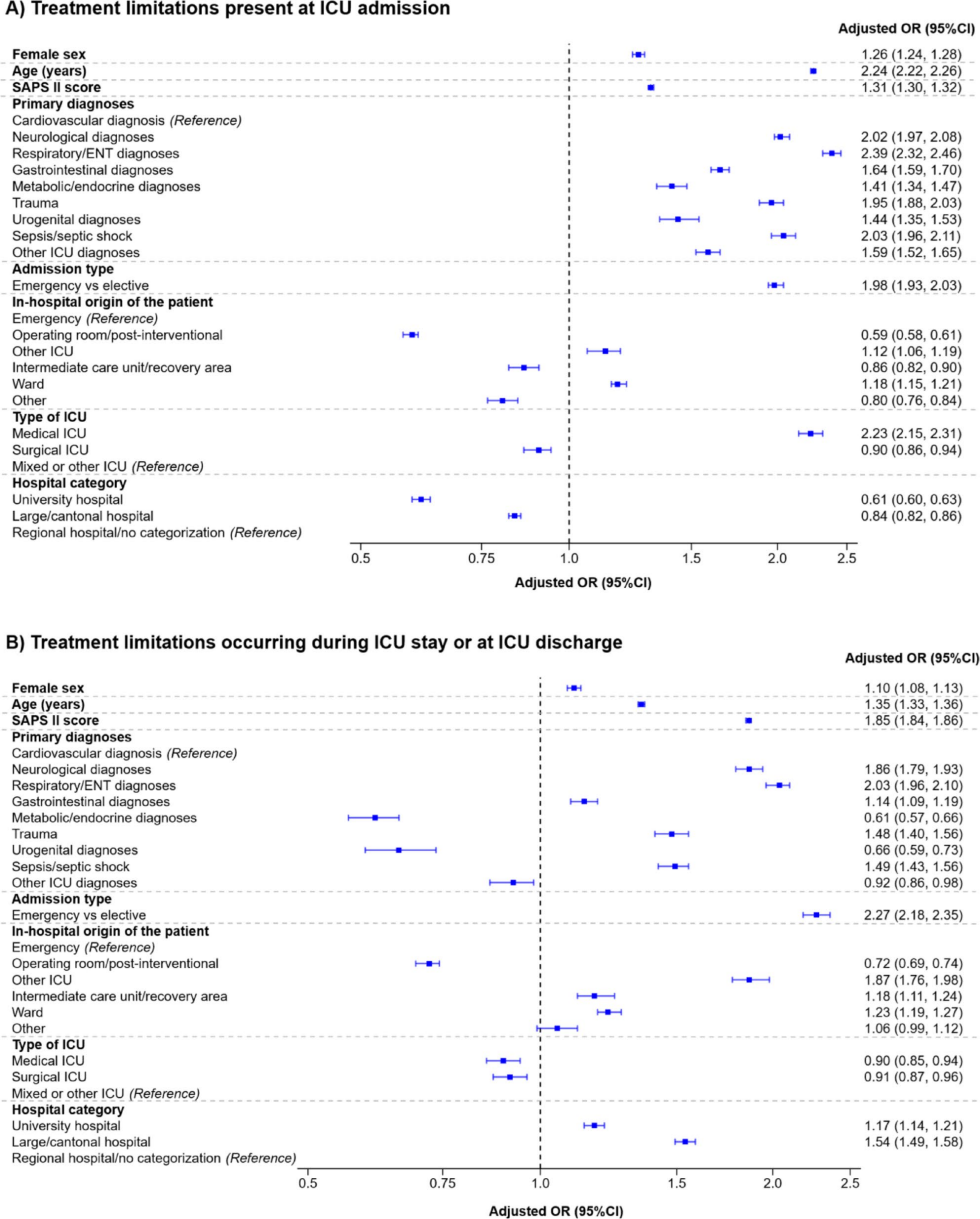
Multivariable association between sex and treatment limitation decisions. **A**. Adjusted odds ratios for treatment limitations present at ICU admission (Model 1). **B**. Adjusted odds ratios for treatment limitations occurring during the ICU stay or at ICU discharge among patients without limitations at ICU admission (Model 2). Estimates are derived from multivariable logistic regression models adjusted for age, illness severity (SAPS II), admission diagnosis category, admission pathway, and institutional characteristics. Odds ratios are shown with 95% confidence intervals. The sex-by-diagnosis interaction model for Model 1 is presented in Supplementary Table 3. CI, confidence interval; ICU, intensive care unit; SAPS II, Simplified Acute Physiology Score II

In the sex-by-diagnosis interaction model (Supplementary Table 3), the magnitude of the sex difference varied across diagnostic categories. Women had a substantially higher likelihood of treatment limitations at ICU admission in trauma (aOR 1.45, 95% CI 1.36–1.56) and cardiovascular diagnoses (aOR 1.40, 95% CI 1.35–1.46). Moderate differences were observed in respiratory (aOR 1.25, 95% CI 1.20–1.31), gastrointestinal (aOR 1.21, 95% CI 1.15–1.27), and neurological conditions (aOR 1.18, 95% CI 1.13–1.23). In contrast, no clear sex difference was observed in metabolic/endocrine disorders (aOR 1.05, 95% CI 0.96–1.14). Across nearly all diagnostic groups, women were more likely than men to have treatment limitations at ICU admission.

### Treatment limitations during ICU stay or at ICU discharge

Among patients admitted without limitations, female sex remained associated with treatment limitations during the ICU stay or at discharge, although the magnitude was smaller (aOR 1.10, 95% CI 1.08–1.13, Fig. 2B). In this model, age and illness severity at ICU admission were the dominant predictors (aOR per 10 years 1.35; per 10 SAPS II points 1.85). Also, the diagnostic category was strongly associated with the occurrence of treatment limitations. Results were unchanged in sensitivity analyses including patient origin prior to hospital admission, with slightly improved model performance (Supplementary Table 2).

### Adjusted absolute probability

The adjusted absolute probability of treatment limitations by sex and decision stage is shown in Supplementary Fig. 1. After covariate adjustment, differences between women and men were largest at ICU admission and markedly attenuated for treatment limitations occurring during the ICU stay.

### Association with mortality

Mortality was substantially higher in patients with treatment limitations than in those without, irrespective of sex (Supplementary Fig. 2). When stratified by timing of treatment limitation, a clear gradient was observed, with the highest mortality in patients with limitations at ICU admission, followed by those with limitations during the ICU stay, and the lowest mortality in patients without limitations (Supplementary Fig. 3A–C). Within each timing category, sex differences in mortality were small and not directionally consistent: Among patients with limitations at ICU admission, mortality was slightly higher in men than in women, whereas among patients with limitations arising during the ICU stay, mortality was slightly lower in men; no clear difference was observed for limitations documented at ICU discharge. These findings are descriptive and reflect unadjusted comparisons within timing strata.

## Discussion

In this nationwide Swiss ICU cohort, sex differences in treatment limitations were primarily observed at ICU admission and were attenuated for decisions occurring later during the ICU stay. Women were more likely than men to have treatment limitations documented at ICU admission after adjustment for age, illness severity, diagnosis, and institutional context. Patients with limitations introduced later during the ICU stay had intermediate age but higher illness severity, suggesting decisions in response to clinical deterioration. The magnitude of sex differences varied across diagnoses and was most pronounced in trauma and cardiovascular conditions. These findings indicate that sex differences are context-dependent and emerge mainly during early goal-setting at ICU admission.

Beyond differences in frequency, treatment limitation decisions also differed in their characteristics. Women more often had limitations defined as ceilings of therapy and decisions documented as reflecting the patient’s wishes, whereas men more frequently underwent withdrawal of life-sustaining therapies and had decisions documented as medical decisions. Mortality showed a clear gradient across treatment limitation timing, being highest in patients with limitations at ICU admission, intermediate in those with limitations during ICU stay, and lowest in patients without limitations. Within strata of treatment limitation timing, sex differences in mortality were small and not directionally consistent, indicating that the observed mortality gradient is primarily driven by the presence and timing of treatment limitation rather than by sex. Together, these findings suggest that sex differences arise predominantly at the stage of initial goal setting rather than during ongoing ICU care. This observation further suggests that differences in mortality largely reflect differences in decision pathways rather than differences in disease severity or treatment response alone.

The timing pattern in this study is clinically relevant. Early limitation decisions are often made under time pressure and prognostic uncertainty, conditions that may increase reliance on heuristics and context-dependent decision-making [13, 14]. Clinicians may also anticipate poorer functional recovery in certain patient groups based on age, frailty, or social context, potentially influencing early goal-setting decisions [15, 16]. These differences should not be interpreted as evidence of bias alone, but may also reflect goal-concordant care, including differences in patient preferences, frailty, social support structures, disease biology, and illness trajectories, which are not fully captured in the registry. Subsequent decisions are taken following evaluation of the patient’s response to intensive care therapy and the acquisition of comprehensive information. Accordingly, these decisions seem to be more strongly influenced by the evolving clinical course [17]. In our study, sex differences decreased after admission, suggesting that decisions become more balanced once objective clinical information accumulates. The characteristics of decisions indicate different routes to treatment limitations, rather than differences in the amount of care provided.

The higher prevalence of existing treatment limitations in women at ICU admission may reflect that women facing severe illness are more likely to have documented preferences limiting aggressive care and may hold more realistic expectations regarding the capabilities of modern intensive care medicine compared with men [18, 19]. Importantly, these findings may also be consistent with goal-concordant care rather than differences in care quality, reflecting variation in patient preferences, frailty, social context, or illness trajectories that are not fully captured in the registry. Men with metastatic cancer disease are also more likely to receive non-beneficial intensive care at the end-of-life [20]. Also, patients in general tend to overestimate the possibilities of modern emergency and critical care, as shown in a representative survey of the Swiss population, in which the survival with a favorable neurological outcome after a sudden cardiac arrest was substantially overestimated [21]. In line with previous research, women more often had predefined ceilings of care, whereas men more often underwent withdrawal of life-sustaining therapies after escalation of treatment [18–20]. Patient wishes as the documented reason for treatment limitations were more frequent among women, whereas decisions among men were more often recorded as medical or physician-driven. These categories should not be interpreted as mutually exclusive, but rather as reflecting the predominant documented driver within a complex interplay between patient preferences, surrogate decision-makers, and physician recommendations, indicating different pathways to treatment limitations rather than unequal care. Interestingly, we did not observe sex differences in the involvement of surrogate decision-makers. This finding may appear unexpected given demographic differences, such as a higher proportion of older women living alone or being widowed. However, surrogate involvement may reflect the availability of broader social networks beyond marital status, including adult children or other relatives, and may also be influenced by institutional practices in identifying surrogate decision-makers. In addition, the registry captures only the presence of surrogate involvement but not the structure or dynamics of the decision-making process, which may differ between women and men.

Prior studies have demonstrated sex differences in ICU treatment intensity and outcomes, including lower use of invasive organ support in women and age- and diagnosis-dependent mortality differences [6, 7, 22]. Registry analyses have similarly reported systematic differences in ICU care processes between women and men [8]. Specifically, female cardiac arrest survivors seem to be at risk for premature withdrawal of life-sustaining therapies within 72 h after cardiac arrest [23, 24]. These observations have remained difficult to interpret because mortality in intensive care frequently follows treatment limitation decisions rather than refractory physiological failure alone [23, 25]. In addition, the timing and characteristics of end-of-life decisions vary considerably across settings [1]. The present analysis suggests a potential explanation: Sex differences appear concentrated at ICU admission - particularly in trauma and cardiovascular conditions - when prognostic uncertainty dominates. Once objective clinical information accumulates during the ICU stay, these differences diminish. This pattern is consistent with prior work suggesting that disparities in critical care are context-dependent rather than uniform across the care pathway [26]. 

These findings have practical implications for ICU care. Variability appears to arise primarily during early goal-setting rather than during the withdrawal process itself. Structured and systematic discussion of treatment goals at ICU admission may therefore be particularly important to ensure consistent decision pathways across patients. The observed differences also emphasize the importance of proactively eliciting and documenting patient preferences [27, 28]. These findings may further suggest a need for earlier and more proactive advance care planning, in primary care and outpatient settings before the onset of critical illness, particularly among male patients. Standardized approaches to advanced care discussions and training on bias may reduce variability in escalation patterns and support goal-concordant care, thereby potentially reducing family distress and treatment intensity at the end-of-life [27, 29]. This is particularly relevant in Europe, where only a minority of intensive care unit patients have documented advance directives [30]. 

The strengths of this study include the large nationwide cohort, mandatory registry reporting, and detailed documentation of treatment limitation timing and characteristics, allowing separation of early triage decisions from later prognosis-based decisions. Several limitations should be considered. First, as an observational study, causal inferences cannot be made, and unmeasured confounding may remain. In addition, we did not explicitly account for clustering at the center level; local institutional practices and culture may influence treatment limitation decisions and could contribute to residual confounding. Second, the registry does not capture detailed information on pre-existing advance directives, patient preferences, comorbidity burden, or validated measures of frailty, all of which are important determinants of treatment limitation decisions and may differ between women and men. Although we included patient origin prior to hospital admission as a proxy for baseline functional status in sensitivity analyses, this measure remains limited and does not fully capture frailty or comorbidity burden. The stability of results after adjustment for this proxy suggests that the observed sex differences are not fully explained by baseline dependency alone. Third, our analysis is restricted to patients admitted to the ICU and does not capture potential sex differences in ICU admission decisions themselves, which may represent an additional layer of selection. Fourth, while sex was used as the exposure variable, the observed differences likely reflect a complex interplay between biological sex and gender-related factors such as communication patterns, health literacy, and advance care planning behaviors, which are not captured in the registry. Finally, although ICU readmissions within 48 h were excluded, repeat admissions beyond this window may still occur and could introduce some degree of non-independence, although this is unlikely to materially bias sex-specific comparisons.

## Conclusions

In this nationwide ICU registry, sex differences in treatment limitations were concentrated at ICU admission and largely attenuated thereafter. Differences in the characteristics of decisions suggest distinct pathways to treatment limitations, with more anticipatory goal-setting in women and more escalation followed by withdrawal in men. These findings indicate that variability arises primarily during early goal definition rather than later management and highlight the importance of structured discussions of treatment goals at ICU admission or in the outpatient setting.

## Supplementary Information

Supplementary Material 1

## Data Availability

This study is based on the MDSi dataset by the Swiss Society of Intensive Care Medicine (SSICM). The availability of this data is subject to restrictions, and any requests must be made to the scientific committee of the SSICM (https://www.sgi-ssmi.ch).
