# Supplementary material for: Timing matters: sex differences in treatment limitation decisions in intensive care
Source: Crit Care. 2026 Jun 19;30:331. doi: 10.1186/s13054-026-06139-x (PMC13307682; doi:10.1186/s13054-026-06139-x)
Supplement: Supplementary file 1 — Supplementary Material 1 [file 13054_2026_6139_MOESM1_ESM.docx]

**Supplementary Material**

**Manuscript:**

**Timing matters: Sex differences in treatment limitation decisions in intensive care**

| **Characteristic** | **Men**  **(n= 395,440)** | **Women**  **(n=259,220)** | **SMD** |
| --- | --- | --- | --- |
| **Timing of limitations** |  |  |  |
| No limitations | 337,532 (85.4%) | 212,205 (81.9%) | 0.094 |
| At ICU admission | 34,031 (8.6%) | 31,129 (12.0%) | -0.112 |
| During ICU stay | 21,698 (5.5%) | 14,365 (5.5%) | -0.002 |
| At ICU discharge | 2,179 (0.6%) | 1,521 (0.6%) | -0.005 |

**A.**

**B.**

| **Characteristic** | **Men**  **(n= 57,908)** | **Women**  **(n=47,015)** | **SMD** |
| --- | --- | --- | --- |
| **Degree of limitations** |  |  |  |
| Patient’s wish | 2,140 (3.7%) | 1,813 (3.9%) | -0.008 |
| Health advocate’s wish | 42,070 (72.6%) | 35,916 (76.4%) | -0.085 |
| Medical decision | 13,698 (23.7%) | 9,286 (19.8%) | 0.095 |
| **Reason for limitations** |  |  |  |
| Time-limited | 27,259 (47.1%) | 25,340 (53.9%) | -0.137 |
| Content-limited | 7,415 (12.8%) | 5,476 (11.6%) | 0.035 |
| Palliation / withdrawal | 23,234 (40.1%) | 16,199 (34.5%) | 0.117 |

**Supplementary Table 1. Timing and characteristics of treatment limitations decisions according to sex. A. Timing of treatment limitations in the overall ICU population.** Percentages are calculated within the overall ICU population. **B. Degree and reason for treatment limitations among patients with documented limitation.** Proportions are calculated within the subgroup of patients with documented treatment limitation and therefore describe decision characteristics rather than the probability of treatment limitations. Values are presented as counts and proportions. ICU, intensive care unit: SMD, standardized mean difference.

|  | **Treatment limitations present at ICU admission** | | **Treatment limitations occurring during ICU stay or at ICU discharge** | |
| --- | --- | --- | --- | --- |
|  | **Adjusted OR [95% CI]** | **p-value** | **Adjusted OR [95% CI]** | **p-value** |
| Female sex vs male sex | 1.25 [1.23, 1.27] | <0.001 | 1.10 [1.08, 1.13] | <0.001 |
| Age (years)* | 2.23 [2.20, 2.25] | <0.001 | 1.35 [1.33, 1.36] | <0.001 |
| SAPS II score* | 1.31 [1.30, 1.31] | <0.001 | 1.85 [1.84, 1.86] | <0.001 |
| **Primary diagnoses** |  |  |  |  |
| Cardiovascular diagnosis | 1.00 (Reference) |  | 1.00 (Reference) |  |
| Neurological diagnoses | 1.98 [1.93, 2.04] | <0.001 | 1.85 [1.79, 1.92] | <0.001 |
| Respiratory/ENT diagnoses | 2.36 [2.30, 2.43] | <0.001 | 2.02 [1.95, 2.09] | <0.001 |
| Gastrointestinal diagnoses | 1.63 [1.58, 1.68] | <0.001 | 1.14 [1.10, 1.19] | <0.001 |
| Metabolic/endocrine diagnoses | 1.39 [1.33, 1.46] | <0.001 | 0.61 [0.57, 0.66] | <0.001 |
| Trauma | 1.86 [1.79, 1.93] | <0.001 | 1.48 [1.40, 1.56] | <0.001 |
| Urogenital diagnoses | 1.41 [1.33, 1.50] | <0.001 | 0.66 [0.59, 0.73] | <0.001 |
| Sepsis/septic shock | 2.00 [1.93, 2.08] | <0.001 | 1.48 [1.42, 1.55] | <0.001 |
| Other ICU diagnoses | 1.57 [1.50, 1.63] | <0.001 | 0.93 [0.87, 0.99] | 0.016 |
| **Admission type** |  |  |  |  |
| Emergency vs elective | 1.96 [1.91, 2.01] | <0.001 | 2.24 [2.16, 2.33] | <0.001 |
| **In-hospital origin of the patient** |  |  |  |  |
| Emergency | 1.00 (Reference) |  | 1.00 (Reference) |  |
| Operating room/post-interventional | 0.59 [0.58, 0.61] | <0.001 | 0.72 [0.69, 0.74] | <0.001 |
| Other ICU | 1.10 [1.04, 1.16] | 0.002 | 1.62 [1.53, 1.73] | <0.001 |
| Intermediate care unit/recovery area | 0.85 [0.81, 0.89] | <0.001 | 1.16 [1.10, 1.23] | <0.001 |
| Ward | 1.17 [1.15, 1.20] | <0.001 | 1.24 [1.20, 1.28] | <0.001 |
| Other | 0.78 [0.74, 0.83] | <0.001 | 0.98 [0.92, 1.04] | 0.569 |
| **Type of ICU** |  |  |  |  |
| Mixed or other ICU | 1.00 (Reference) |  | 1.00 (Reference) |  |
| Medical ICU | 2.25 [2.17, 2.34] | <0.001 | 0.89 [0.84, 0.93] | <0.001 |
| Surgical ICU | 0.93 [0.89, 0.97] | 0.001 | 0.90 [0.86, 0.95] | <0.001 |
| **Hospital category** |  |  |  |  |
| Regional hospital/no category | 1.00 (Reference) |  | 1.00 (Reference) |  |
| University hospital | 0.60 [0.58, 0.62] | <0.001 | 1.17 [1.13, 1.20] | <0.001 |
| Large/cantonal hospital | 0.84 [0.82, 0.86] | <0.001 | 1.53 [1.48, 1.57] | <0.001 |
| **Pre-hospital patient origin** |  |  |  |  |
| Other | 1.00 (Reference) |  | 1.00 (Reference) |  |
| Home | 0.81 [0.79, 0.84] | <0.001 | 0.98 [0.95, 1.02] | 0.345 |
| Transfer from other hospital | 0.87 [0.84, 0.91] | <0.001 | 1.27 [1.21, 1.33] | <0.001 |
| Nursing facility | 2.44 [2.27, 2.62] | <0.001 | 1.52 [1.36, 1.70] | <0.001 |
| **Number of observations** | 614897 |  | 589500 |  |
| **BIC** | 322269.85 |  | 213169.92 |  |

**Supplementary Table 2. Multivariable models including patient origin prior to hospital admission.**
Multivariable logistic regression analyses assessing the association between sex and treatment limitation decisions, additionally adjusted for patient origin (home, nursing facility, or transfer) as a proxy for baseline functional status. Models are shown for treatment limitation at ICU admission (left) and treatment limitation during ICU stay (right). Results are presented as adjusted ORs with 95% CIs. Abbreviations: BIC, Bayesian Information Criterion; CI, confidence interval ; ENT, ear-nose-throat; ICU, intensive care unit; OR, odds ratio; SAPS II, Simplified Acute Physiology Score II. *Adjusted ORs are reported per 10-unit increase for age and SAP II score.

| **Primary diagnosis category** | **Female vs male: Adjusted OR [95% CI]** |
| --- | --- |
| Trauma | 1.45 [1.36, 1.56] |
| Cardiovascular diagnoses | 1.40 [1.35, 1.46] |
| Respiratory and ENT diagnoses | 1.25 [1.20, 1.31] |
| Gastrointestinal diagnoses | 1.21 [1.15, 1.27] |
| Neurological diagnoses | 1.18 [1.13, 1.23] |
| Urogenital | 1.18 [1.05, 1.34] |
| Other ICU diseases | 1.17 [1.09, 1.26] |
| Sepsis/Septic shock | 1.16 [1.09, 1.24] |
| Metabolic/endocrine diagnoses | 1.05 [0.96, 1.14] |

**Supplementary Table 3. Sex-by-diagnosis interaction for treatment limitation at ICU admission.** Adjusted odds ratios (OR) and 95% confidence intervals (CI) from a logistic regression model assessing treatment limitation at ICU admission compared with no limitation (n = 614,897). The model includes an interaction term between sex and primary diagnosis category to evaluate effect modification. All models were adjusted for age (per 10 years), SAPS II score (per 10 points), admission type, in-hospital origin, ICU type, and hospital category. Abbreviations: CI, confidence interval; ENT, ear, nose and throat; ICU, intensive care unit; OR, odds ratio; SAPS II, Simplified Acute Physiology Score II.


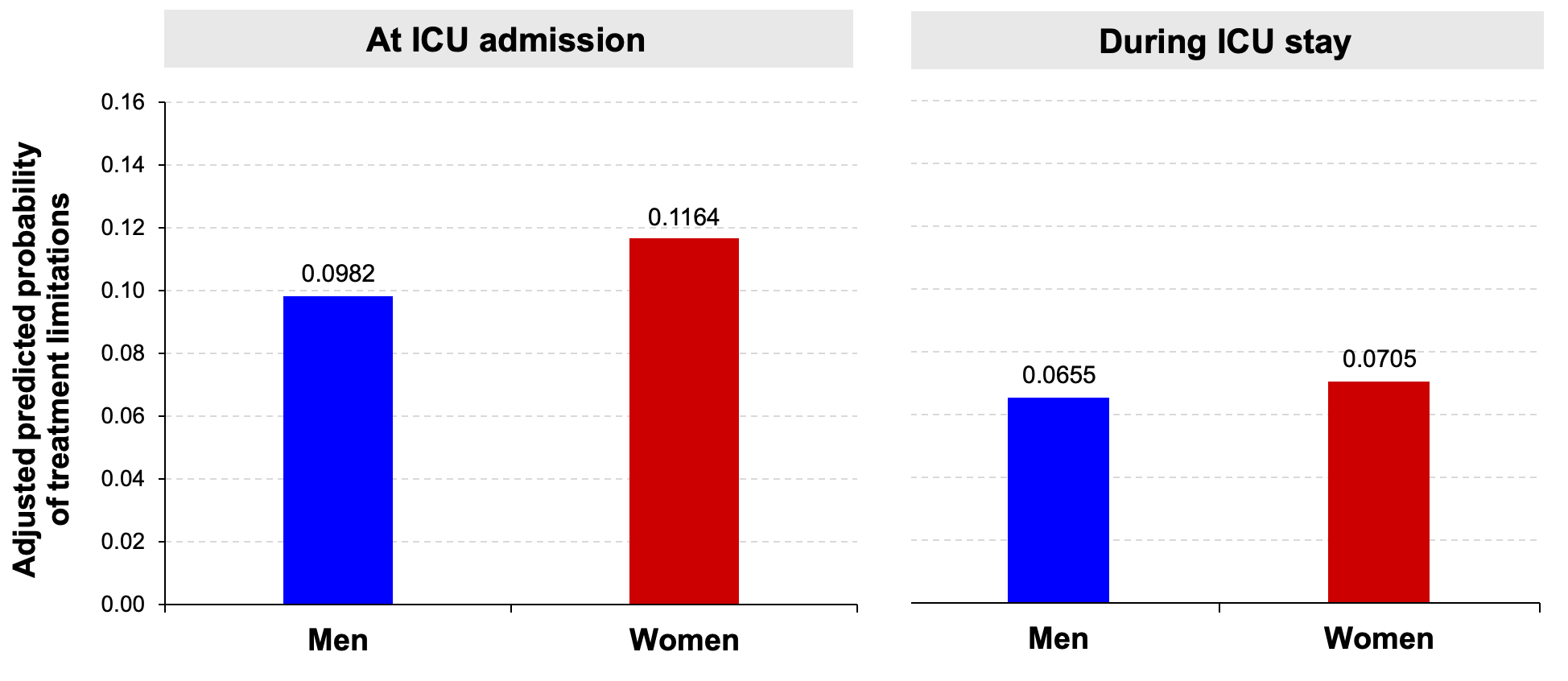


**Supplementary Figure 1. Adjusted predicted probability of treatment limitation by sex and decision stage.** Predicted probabilities derived from multivariable models standardized to the covariate distribution of the study population.

**
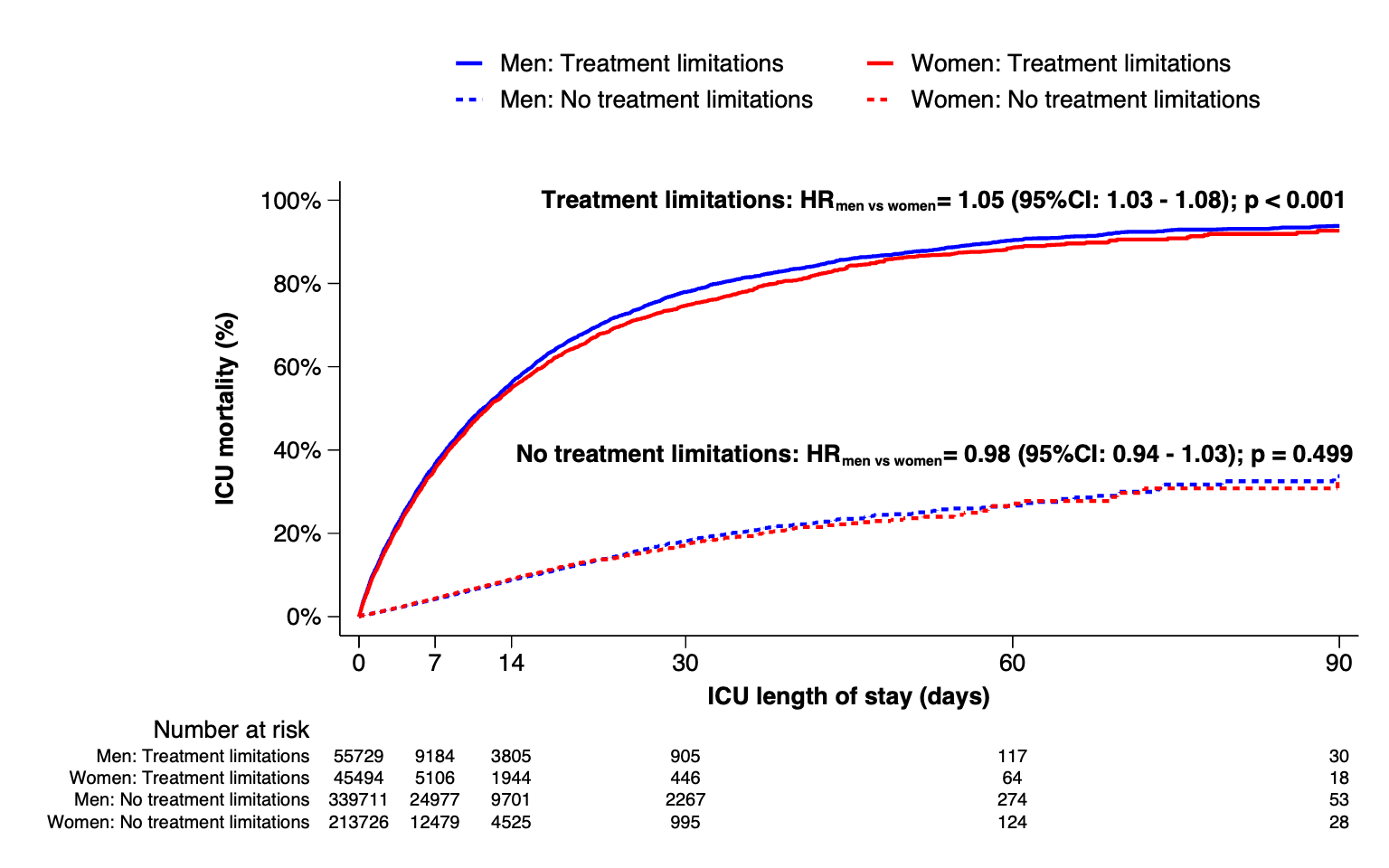
**

**Supplementary Figure 2. ICU mortality according to treatment limitation status.** Kaplan–Meier estimates of ICU mortality in patients with and without documented treatment limitations, shown separately for women and men. Curves represent unadjusted cumulative mortality over ICU length of stay and describe outcome according to limitation status rather than a causal effect of treatment limitations. Abbreviations: CI, confidence interval; ICU, intensive care unit; HR, hazard ratio


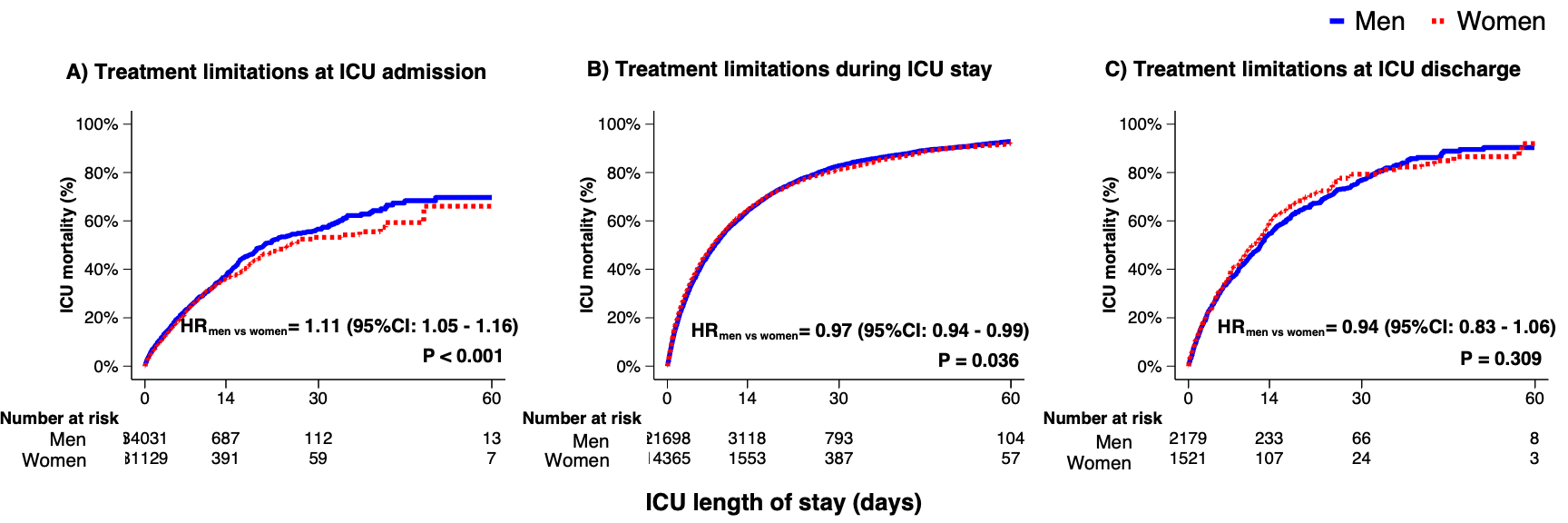


**Supplementary Figure 3. ICU mortality according to timing of treatment limitation and sex.** Kaplan–Meier estimates of ICU mortality stratified by sex within timing categories of treatment limitation. (A) limitation present at ICU admission, (B) limitation occurring during the ICU stay, and (C) limitation documented at ICU discharge. Curves represent unadjusted comparisons between women and men within each stratum; hazard ratios correspond to unadjusted Cox models. Percentages indicate cumulative mortality over ICU length of stay. Abbreviations: CI, confidence interval; ICU, intensive care unit; HR, hazard ratio.
